# Occurrence of Magellanic Penguins along the Northeast Brazilian Coast during 2008 Austral Winter

**DOI:** 10.1100/2012/686184

**Published:** 2012-05-02

**Authors:** Renato Ramos da Silva, Janini Pereira, Clemente A. S. Tanajura, Carlos A. D. Lentini, Mauro Cirano, P. Dee Boersma, Regina R. Rodrigues

**Affiliations:** ^1^Universidade Federal de Santa Catarina, Campus Trindade, Florianópolis, SC, 88040-900, Brazil; ^2^Grupo de Oceanografia Tropical, Instituto de Física, Universidade Federal da Bahia, Salvador, BA, 40170-280, Brazil; ^3^Aquatic Sciences, South Australian Research and Development Institute, Adelaide, SA, 5024, Australia; ^4^Department of Biology, University of Washington, Seattle, WA, 98195-1800, USA

## Abstract

During the austral winter of 2008, thousands of penguins traveled to low latitudes along the South Atlantic coast of South America. The atmospheric and oceanic conditions from April to July 2008 may account for the penguins' unusual geographic distribution. During that period, South Atlantic coastal waters were cooler; the wind anomalies had northward and onshore components; the ocean's coastal region presented northward currents that favored the penguins to travel toward lower latitudes. This anomalous climate regime resulted from extreme meteorological frontal systems that occurred mainly during June 2008. Three consecutive extreme midlatitude cyclones produced strong wind shear that resulted in the northward oceanic flow along the South American eastern shoreline favoring the penguins to be spotted in northern tropical waters.

## 1. Introduction

The Magellanic penguins (*Spheniscus magellanicus*) breed from about 40°S latitude south in Chile, Argentina, and the Malvinas (Falkland) Islands [1]. Penguins are central-place foragers while raising chicks but after reproduction in Argentina they leave their breeding colonies traveling north where their prey is more abundant [2, 3]. These penguins swim at the surface and can travel up to 173 km per day [3]. In the South Atlantic Ocean, Magellanic penguins face problems from oil pollution, overfishing, to climate variation [4].

The ongoing climate change is affecting the entire planet [5]. The consequence of this impact on the biodiversity is of great concern. For example, the emperor penguin of Antarctica is strongly dependent on the ice cover, and recent climate change is affecting their population [6, 7]. The South Atlantic experienced an extreme event in March 2004, the first time a hurricane was recorded in this region [8, 9]. Understanding the regional climate change and the occurrence of extreme events and their impacts on marine populations remains an important issue [5].

**Figure 1:**
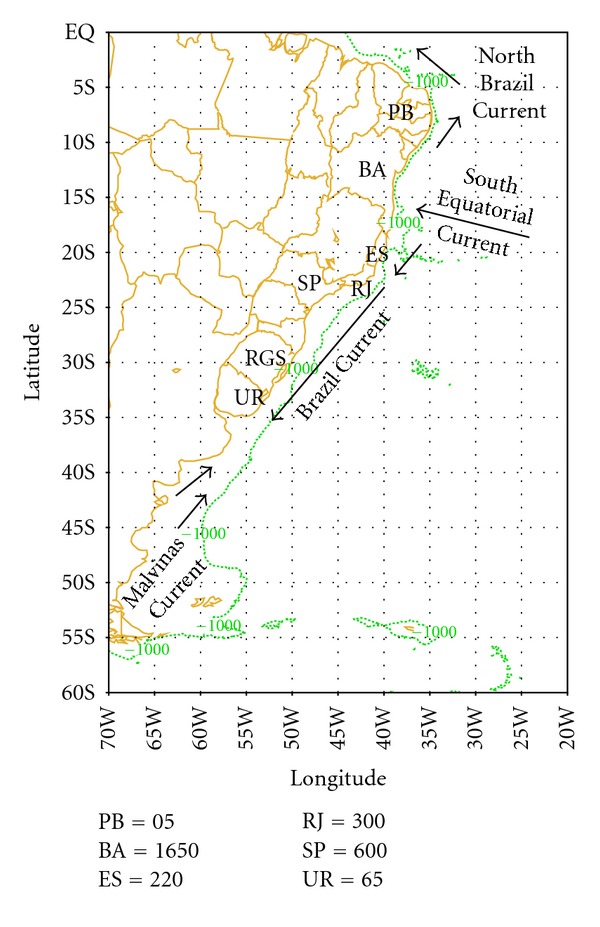
Schematic representation of South America coastal ocean currents and locations representing the following: UR: Uruguay, RGS: Rio Grande do Sul, SP: São Paulo, ES: Espírito Santo, BA: Bahia, PB: Paraiba, and S is the city of Salvador, BA. The numbers at the left corner represent the total of penguins that arrived at those locations. The green line shows the 1000 m isobath. Adapted from [10].

The area of this study, which encompasses the western South Atlantic Ocean, is characterized by at least four different hydrographic regimes: (i) the bifurcation of the South Equatorial Current (SEC), (ii) the Brazil Current (BC), (iii) the Malvinas Current (MC), and (iv) the Brazil-Malvinas Confluence (BMC) region (Figure 1).

The region where the SEC bifurcates is not steady and is expected to have a meridional seasonal migration. Recent studies show that the SEC bifurcation reaches its southernmost position in July (i.e., ~17°S) and its northernmost position in November (i.e., ~13°S), which is associated to changes in the local wind due to the annual north-south migration of the Intertropical Convergence Zone (ITCZ) [11]. At the bifurcation region, three branches of the eastward flowing SEC approach the eastern coast of South America between 7° and 17°S [12, 13]. The northern and central branches are related to the origin of the North Brazil Current (NBC), whereas the southern branch feeds the BC at the surface [10].

The southward flow of tropical waters from the BC is characterized by a relatively weak and shallow current when compared to other major Western Boundary Currents and is basically confined to the shelf break between 11° to 36°S, where it separates from the coastline and veers offshore [10, 14–17].

The northward flow of cold sub-Antarctic waters of the MC is originated as a meander branch of the Antarctic Circumpolar Current [10]. Like the BC, the MC also separates from the continental shelf and slope region at 38°S, and then moves offshore [17].

On annual time scales, the variability of the southwestern portion of the South Atlantic subtropical gyre is dominated by seasonal displacements of the BMC, although many studies have shown that the BMC varies on other nonseasonal time scales [18–21]. The nonseasonal variability is linked to changes in the BC and MC separation creating a strong frontal zone and one of the most energetic regions of the world ocean, which in turn, dictates the position and geometry of the confluence [10, 15, 17, 22, 23]. The BC separation latitude from the coastline is not in general spatially coincident with the separation of the MC, but typically there is a band of surface waters of intermediate temperature of 100 km up to 320 km wide on average between these two opposing flows. The BMC is characterized by strong mesoscale activity, Sea Surface Temperature (SSTs), and chlorophyll gradients [15, 21, 24, 25] that are associated with mechanisms of ocean-atmosphere interaction and water mass formation [23, 26].

At the confluence region, the Río de la Plata waters form a low-salinity buoyancy-driven tongue that affects the inner and midshelf circulation, stratification, and the distribution of nutrients and biological species over a wide extent of the adjacent continental shelf [27, 28]. This northward intrusion of relatively fresh and cool waters from the la Plata region onto the shelf has been suggested as responsible for the recruitment failure of the Brazilian sardine [29, 30] and for the intrusion of organisms from different biogeographical zones, such as the detection of cold water foraminifera, ostracoda, and micro-bivalves, as well as the anecdotal reports of occasional occurrence of subantarctic dolphins and penguins at latitudes lower than 21°S [28, 31]. This low-salinity buoyancy-driven plume shows a seasonal meridional displacement which is in agreement with the seasonal prevailing winds, reaching lower latitudes (28°S) during austral winter associated with southwesterly winds, while during austral summer the dominant northeasterly winds force the plume to retreat to higher latitudes (32°S) [32]. Furthermore, recent studies show that the meridional migrations of the Plata plume are regulated by the along-shore component of the wind stress forcing [33].

From June to September 2008, thousands of penguins reached the shores of Brazil. Monitoring estimates from several biology marine organizations suggest that more than 3000 penguins reached the shores during that austral winter [34]. Several local newspapers reported large number os penguins coming ashore. The Brazilian newspaper *Folha de São Paulo* (see online version at http://www.folha.uol.com.br/) reported the following numbers obtained from several marine animals care centers. Initially, on 08 June 2008, about 65 penguins, many already dead, reached the Uruguayan coast. On 25 June 2008, the first couple of penguins arrived at the coast of the state of São Paulo in Brazil. Then, for the period between 17 and 31 of July, a total of 200 penguins reached the coast of São Paulo state, 220 reached the state of Espírito Santo, and 370 reached the city of Salvador in the state of Bahia, which is located at 13°S. Further on, during September, a total of 14, 26, and 05 penguins reached the states of Alagoas, Sergipe, and Paraiba, respectively. Overall, about 600 and 300 individuals reached the states of São Paulo and Rio de Janeiro, respectively. The majority of these penguins were juveniles with ages varying between 03 months and one year old. Out of the total of 1650 penguins that reached the state of Bahia, only 04 were adults. Figure 1 shows an illustration of the penguin's arrival locations.

A penguin rehabilitation center (*Instituto do Meio Ambiente-IMA*) in the city of Salvador-BA took care of several weak and ill juveniles. On 3rd of October 2008, 399 recovered penguins were transported from Salvador to the southern state of Rio Grande do Sul in a Hercules C-130 airplane by the Brazilian Air Force. The penguins were released at the Rehabilitation Center for Marine Animals (CRAM) located at the city of Rio Grande-RS (32°S) on the 4th of October 2008. The four adults were expected to guide the more inexperienced juveniles back home.

Although there is some evidence that subantarctic species can be found in subtropical regions up to 21°S [28], this is one of the first times subantarctic penguins were found at latitudes lower than 10°S in tropical waters of Brazilian shorelines. Therefore, why did the penguins travel so far north? Three possible hypothesis arise: (i) the penguins traveled to find food; (ii) anomalous oceanic and climate conditions pushed them farther north; (iii) or both, where the lack of food and currents moved them north.

We performed analysis of oceanic and atmospheric conditions, and an oceanic model simulation, to investigate possible environmental effects on the northern migration of Magellanic penguins. From the aforementioned discussion, our purpose was to identify the atmospheric and oceanic mechanisms responsible for the occurrence of Magellanic penguins in tropical latitudes north of 21°S.

## 2. Material and Methods

To characterize the climate conditions for the period between April and July of 2008, we SSTs, wind, and ocean current fields from observations and numerical outputs. We used SST anomaly fields from the U.S. National Oceanic and Atmospheric Administration (NOAA) Optimal Interpolation dataset, which estimates SSTs based on in situ and satellite retrievals plus SSTs simulated by sea ice cover [35]; atmospheric fields from the National Center for Environmental Prediction/National Center for Atmospheric Research (NCEP/NCAR) reanalysis [36] were also used in conjunction with the ocean currents from the OSCAR (Ocean Surface Current Analysis-Real Time), which provides near real-time ocean velocities fields from the altimeter satellite and vector wind data [37].

Furthermore, the hybrid coordinate ocean model (HYCOM) [38–40] was used to simulate the oceanic conditions for that period. The model was implemented for the South Atlantic region covering the domain from latitudes 45.18°S to 10.20°N and longitudes 68.00°W and 18.00°W with an eddy-resolving horizontal resolution of 0.08°. In the vertical, the model has 21 hybrid levels with higher resolution at the surface mixed layer. The model was run for a period of 40 years forced with climatological atmospheric fields in order to reach an equilibrium state. Then, the model was run for the year of 2008 forced by the 6-hourly NCEP/NCAR reanalysis atmospheric fields of radiation balance, surface air temperature and moisture, precipitation, and wind momentum fluxes. With the model we investigate the air-sea interaction during strong weather events, mainly during June of 2008, because this is when the penguins travel to northern latitudes.

## 3. The Atmospheric and Oceanic Conditions during 2008

**Figure 2:**
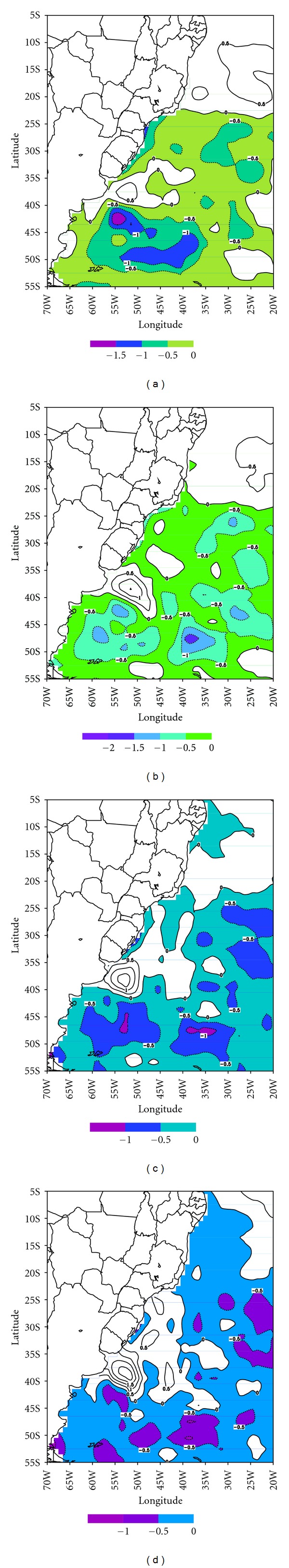
SST anomaly from NOAA-OI for 2008 relative to the mean from 1982 to 2008 for (a) April, (b) May, (c) June, and (d) July (source: http://www.emc.ncep.noaa.gov/).

 Monthly fields of SST anomalies have showed that the South American eastern coastline was cooler during the period between April and July of 2008 (Figure 2). During April, SST anomalies were cooler for most of the coastal region, reaching up to −1°C in several areas mainly in the southern regions and along the coastal waters of southern Brazil (Figure 2(a)). During May, this cooler anomaly remained over most of the region (Figure 2(b)). During June, negative SST anomalies expanded over lower latitudes [5–10°S] (Figure 2(c)), while during July this cooler anomalous surface pattern remained for most of region covering the entire northeast coast of Brazil (Figure 2(d)).

**Figure 3:**
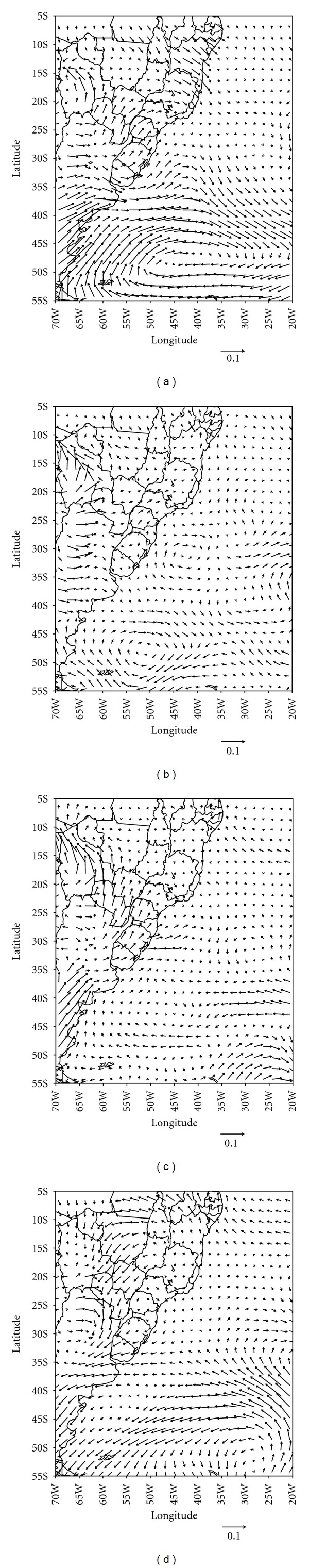
Wind anomaly from NCEP/NCAR reanalysis relative to the mean from 1998 to 2008 for (a) April, (b) May, (c) June, and (d) July.

 During the same period, wind anomalies show anomalous atmospheric circulation patterns that suggest northward components over several regions (Figure 3). During April, the most predominant wind anomaly was northward near the coast between 27 and 30°S. During May, these northward anomalies expanded for the entire coastal areas. During June, the northward wind anomalies remained in the region but onshore anomalies were predominant at the latitudes between 13° and 27°S. During July, southward anomalies were presented at the southern latitudes, but the northward wind anomalies remained at the tropical latitudes (Figure 3(d)).

 The ocean transport integrated within the mixed layer (approximately top 50 m) is given by the Ekman drift which is 90° to the left of the wind direction in the Southern Hemisphere. Although for a northward wind anomaly, the transport along the coast would be onshore. At the surface, where the penguins swim (~10 m), the ocean currents are almost in the same direction of the wind. This is corroborated by the ocean model results.

**Figure 4:**
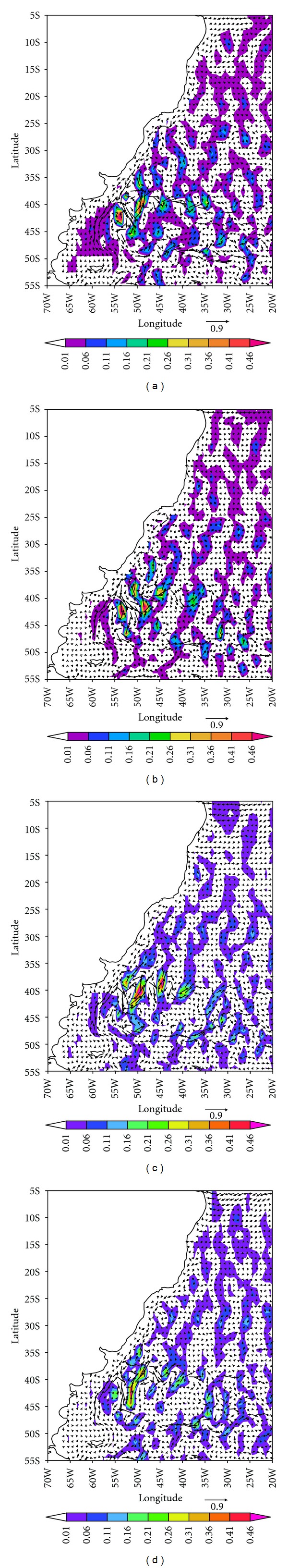
Ocean currents and positive meridional velocity anomaly (ms^−1^) relative to the mean from 1993 to 2009 from the program OSCAR, for (a) April, (b) May, (c) June, and (d) July (source: http://www.oscar.noaa.gov/).

 Mean ocean currents and their anomalies showed northward flow on several regions of the South Atlantic. In the south, the surface velocities were stronger than average in April (Figure 4(a)). For the period between April and July, there were several anomalous northward corridors (Figure 4). At that time, northward surface currents were observed near the coast mainly around 40°S. In June, these northward currents extended to lower-latitudes around 30°S and they could have favored the penguin locomotion to lower latitude regions.

**Figure 5:**
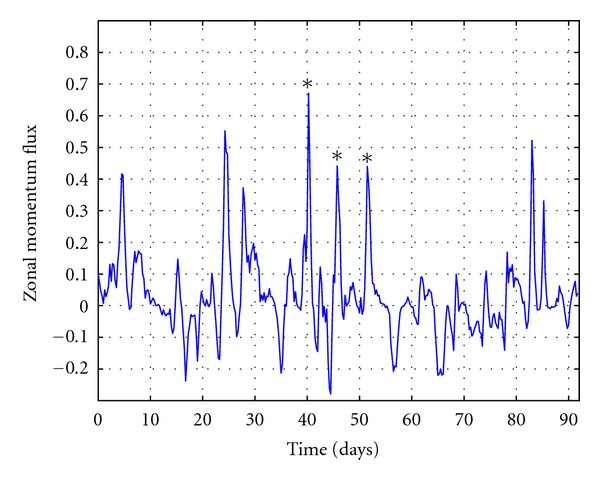
Meridional momentum flux from NCEP/NCAR Reanalysis dataset at 50°W, 36°S. The stars highlight the three consecutive strong fronts that reached the location on June 2008 for 10th, 16th, and 22nd.

 The HYCOM model simulation showed that the strong air-sea interaction was responsible for the major environmental changes during the period. Strong mean latitude winter cyclones moved over the South Atlantic producing the anomalous air-sea changes. In particular, three consecutive cyclones occurred in June only few days apart.  Figure 5 shows the NCEP-reanalysis meridional component of the momentum fluxes at 50°W and 36°S, near the southern coast of Brazil. These cyclones crossed the area around 10th, 16th, and 22nd of June.

**Figure 6:**
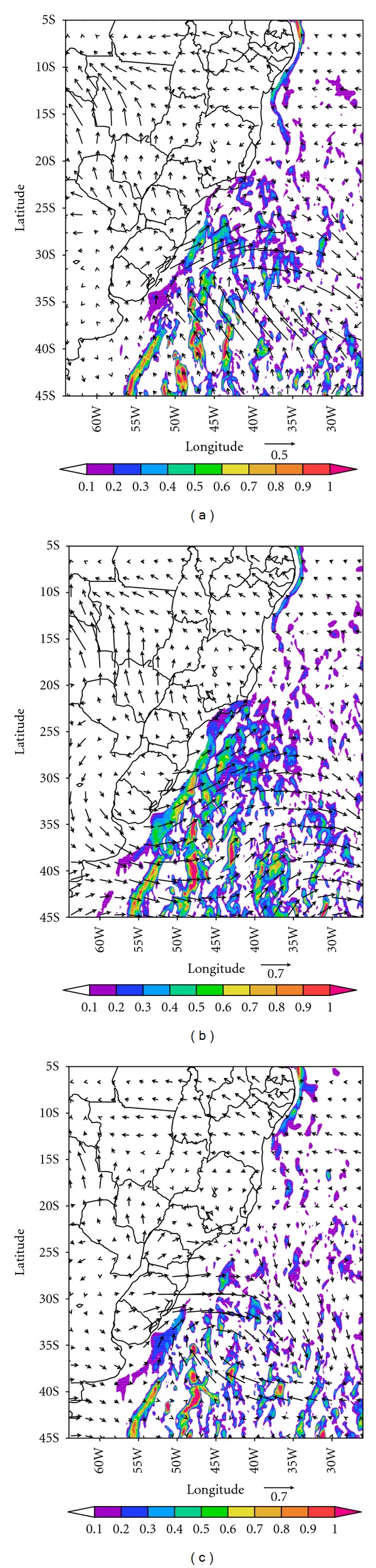
Snapshots for atmospheric momentum flux vectors from NCEP Reanalysis, and HYCOM northward meridional ocean currents (shaded) for (a) 10th, (b) 16th, and (c) 22nd of June 2008.

Figure 6 shows combined maps of NCEP reanalysis momentum fluxes used to force the HYCOM oceanic model and the simulated northward oceanic transport. This figure shows that the strong atmospheric forcing resulted from the migration of the cyclones that produced northward oceanic corridors near the coastal areas of eastern South America. The near-coastal corridors may drive this northward coastal current that had been observed in some cases during austral winter [27, 31, 41]. At this time of the year, the BMC position moves northward around 30–35°S. At the same time, the SEC bifurcation is in its southernmost position [11], and as a consequence the northward transport of the NBC is present near the Brazilian northeast coast around 15°S (Figure 6). Over half of the dives that penguins make are within the top first 10 meters or less, where they remain submerged usually less than 2 minutes [42]. Hence Magellanic penguins spend most of their time near or on the surface of the ocean. Penguins may have traveled north taking advantage of this northward ocean current.

## 4. Discussion

 Environmental climatological analysis for the period between April and July of 2008, showed that SSTs along the South American eastern coastline were coastal were cooler than average and winds and oceanic currents had anomalous northward flows. In the first months of 2008 the Pacific Ocean near near the western coast of South America was cooler than average, which characterizes La Niña conditions, but in the following months, the Pacific returned to normal conditions. However, the Atlantic Ocean was very anomalous. Since the South Atlantic was cooler and the North Atlantic was warmer, the Atlantic SST dipole, coined by Servain [43], was positive for most of 2008. These anomalous climate conditions likely influenced the penguin's unusual travel to lower latitudes.

The meteorological and oceanic conditions for the period were a consequence of consecutive extreme cyclone events. The combination of these events with the penguins' northward migration in search for food may have pushed them farther north than in other years. The cooler climate conditions suggest that the penguins extended their northern migration toward tropical latitudes.

**Figure 7:**
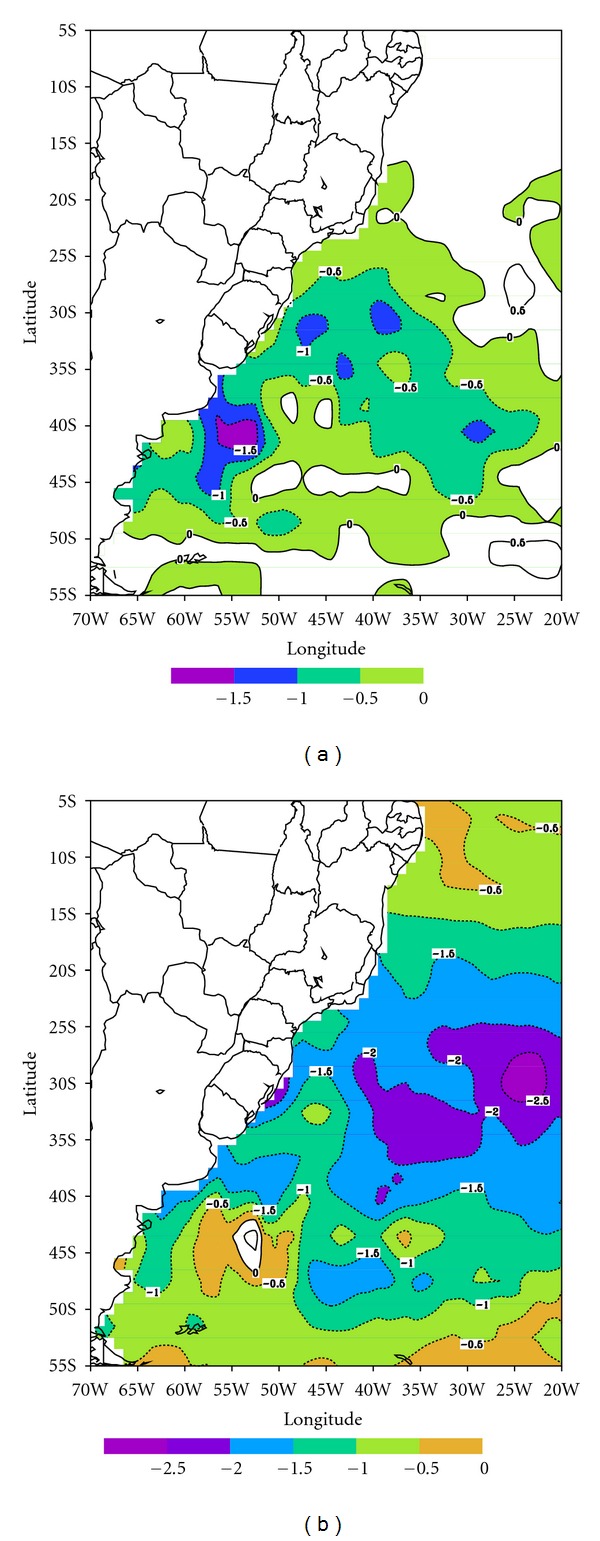
SST anomaly from NOAA-OI for June 1985 (a) and 2000 (b) relative to the mean from 1982 to 2008.

While the year of 2008 had unusual high number of penguins reaching very low latitudes in the northern regions of Brazil, their arrival in the southern coast is common. For instance, during the winter of 1985 and 2000 several penguins were reported to reach the shores of Brazil (source: Brazilian newspapers). A common feature in these two cases is that SST was anomalously cooler (Figure 7). Thus climate variability shows important effects on the penguin's behavior with considerable impacts on their population. Thus, a climate monitoring along with the ecosystems continuous watch should provide important insights into their interactions.
